# Effect of *Artemisia rupestris* L. Extract on Gastrointestinal Hormones and Brain-Gut Peptides in Functional Dyspepsia Rats

**DOI:** 10.1155/2020/2528617

**Published:** 2020-11-16

**Authors:** Chun Wang, Bin Wang, Maimaiti Aili, Shixia Huo, Meng-ting Han, Silafu Aibai, Zhi-Jian Li

**Affiliations:** ^1^Department of Toxicology Laboratory, Xinjiang Institute of Traditional Uygur Medicine, Urumqi, Xinjiang, China; ^2^College of Pharmacy, Xinjiang Medical University, Urumqi, Xinjiang, China; ^3^People's Hospital of Xinjiang Uygur Autonomous Region, Urumqi, Xinjiang, China

## Abstract

*Artemisia rupestris* L. is the perennial herb of rupestris belonging to Artemisia (Compositae), which is wildly distributed in Xinjiang (China), middle Asia, and Europe. It is known to have anti-inflammatory, hepatoprotective, immune function regulation, and gastrointestinal function regulation effects. AR is used to treat digestive diseases, but the effects of AR on antifunctional dyspepsia (FD) activity have not yet been reported. In this study, we aimed to investigate the therapeutic effects of *Artemisia rupestris* L. extract (ARE) on gastrointestinal hormones and brain-gut peptide in functional dyspepsia (FD) rats. Sixty Sprague-Dawley rats were randomly divided into 6 groups. An FD rat model was established by irregular tail clamp stimulation for 14 days except the blank group. After FD rat models, the blank group and model group were given menstruum, and the medicated rats were given corresponding medicine for 14 days. The general observations, bodyweight, and food intake were observed, and the content of serum gastrin (GAS), plasma motilin (MTL), plasma vasoactive intestinal peptide (VIP), and plasma somatostatin (SS) by the enzyme-linked immunosorbent assay was observed. The content of plasma VIP and plasma SS in the ARE group was significantly lower than in the model group, and the content of serum GAS and plasma MTL was increased in the ARE group; the GAS expression of antrum and hypothalamus was increased in the ARE group, and SS expression of antrum and hypothalamus was decreased in the ARE group by immunohistochemical detection; the results of semiquantitative reverse transcription polymerase chain reaction (RT-PCR) indicate that ARE inhibits the mRNA expression of VIP. Our results suggest that ARE can recover gastrointestinal hormone levels and regulation of the peripheral and central nervous system and alter gut peptide levels, which confirm the therapeutic effect of ARE on functional dyspepsia.

## 1. Introduction

Functional dyspepsia (FD), also known as nonulcer dyspepsia, involves upper abdominal pain, persistently recurrent episodes of upper abdominal bloating, early satiety, anorexia, nausea, and abdominal discomfort [1]. Existing studies have shown that the prevalence of FD ranges from 9.8% to 40% in Western populations and 5.3%–28% in Eastern populations [2]. The global prevalence of uninvestigated dyspepsia has reached almost 21% [3]. In the Asian population, the majority of undiagnosed dyspepsia patients without alarm symptoms are diagnosed with functional dyspepsia after gastroscopy [4]. A proportion of 20%–25% of the patients with severe and refractory FD symptoms also have psychosocial comorbidities such as anxiety, depression, or somatization and severely impaired daily functioning [5].

FD is a multifactorial disease with a complex underlying pathophysiology, which may be caused by gastrointestinal disorders, gastrointestinal hormone disorders, reduced sensitivity, *H. pylori* infection, psychological factors, and other symptoms [6]. Furthermore, the digestive tract does not operate independently but instead requires functional integration with other organ systems. A large number of studies have shown that there is continuous communication between the gastrointestinal tract and the nervous system. This communication is bidirectional and is affected by the autonomic nervous system, the immune system, and the hypothalamic-pituitary axis [7]. The complex communication mechanism of the brain-gut axis plays a vital role in regulating body functions and has become the key to understanding disease mechanisms. Gastrointestinal hormones, as an important signal transmission medium in the brain-gut axis, may not only reach the brain directly through the bloodstream but also interact with the nervous system and other pathways to complete brain-gut communication [8].

The main types of drugs currently used to treat FD are proton pump inhibitors (PPIs) and other acid-suppressive agents, prokinetic agents, neuromodulators targeting visceral hypersensitivity, probiotic agents, and herbal therapies [9]. The complexity of the mechanisms has led to a lack of effective targeted therapies, so herbal medicines are starting to come more into view. Compared with single-receptor drugs, herbal medicine can exert a wider range of pharmacological effects. In addition, most patients believe that they have lower side effects although this has not been verified [10]. For example, traditional Chinese medicines such as Xiaoerfupi [11], Fructus aurantii [12], and Sini-San [13] have all been shown to be effective in the treatment of FD.

Chemical composition of *Artemisia rupestris* L. (AR) is mainly keto acids, flavonoids, coumarins, glycosides, etc. [14]. Many studies found that it has tumor inhibition [15], suppressing allergic reactions [16], hepatoprotective [17], anti-inflammatory [18], regulation of immune function [19], and regulation of gastrointestinal function effects [20, 21]. AR is often used to treat functional dyspepsia symptom in Uygur traditional Chinese medicine, but surprisingly, the mechanism of AR's antifunctional dyspepsia (FD) activity has not been reported.

In this study, we aimed to study the pharmacological effects and corresponding changes in gastrointestinal hormones and brain-gut peptides in FD rats after applying *Artemisia rupestris* L. extract (ARE), especially the indexes related to FD including routine parameters such as bodyweight, food intake, serum GAS and plasma MTL, VIP, and SS. Expression of GAS, SS, and VIP in the hypothalamus and gastric antrum was also evaluated. With this, the mechanisms of antifunctional dyspepsia of rats treated with ARE were clarified.

## 2. Materials and Methods

### 2.1. Chemicals

Domperidone (Motilium) was purchased from Xian Janssen Pharmaceutical Co., Ltd., (Beijing, China) under batch number H10910003, and the enzyme-linked immunosorbent assay (ELISA) kits detect for rat GAS were purchased from Nanjing Jiancheng Technology Co., Ltd., (Nanjing, China). The ELISA kits used to detect rat MTL, VIP, and SS were purchased from Shanghai Shengke Bio-Technology Co., Ltd., (Shanghai, China). Aprotinin (20000 U/ml) was purchased from Livzon Pharmaceutical Co., Ltd., (Guangdong, China). GAS antibody and SS antibody were purchased from Wuhan Boster Biological Technology Co., Ltd., (Wuhan, China). Citric acid antigen repair solution, the DAB kit, and phosphate-buffered saline (PBS) were purchased from Beijing Zhongshan jinqiao Biological Co., Ltd., (Beijing, China). BD 2000 was purchased from Swedish Phamacira. Primers for genes were synthesized (Sangon Biotech, Shanghai, China) using the sequence given in Table 1.

### 2.2. Drug Extraction

The AR material was precisely weighed at 100 g and extracted with 8 times the volume of water heated refluxed for 1 h. The filtrates from three extractions were combined. Finally, a water extract containing 0.6 g/mL of the original drug was prepared by concentrating by rotary evaporation..

### 2.3. Animals

Kunming mice (KM) (*n* = 30), male, age: 5-week-old, weight: 18–22 g, clean grade, were obtained from the Xinjiang Laboratory Animal Center. Animals were observed for 3 days after quarantine and were given ordinary feed to make them adapt to the breeding environment. After 3 d, they were randomly grouped according to the experimental requirements.

Male Sprague-Dawley (SD) rats (*n* = 60), weight 180–220 g, were obtained from the Xinjiang Laboratory Animal Center. The animals were housed in the clean animal room at the Center for Evaluation of Drug Safety of Xinjiang Institute of Traditional Uygur Medicine under normal light and dark conditions. The protocols for the experiments were approved by the Xinjiang Institute of Traditional Uygur Medicine Ethics Committee on animal experimentation (approval code: nGLP-YZH-11-006) and conducted in accordance with internationally accepted principles for the use and the care of laboratory animals.

### 2.4. Experimental Design

**Figure 1:**
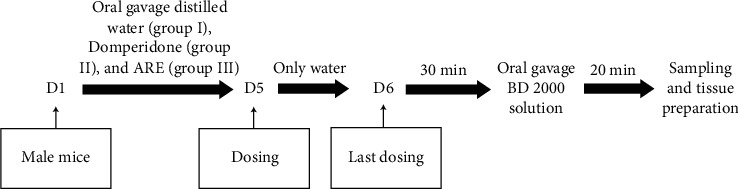
Experimental procedures and time line: each group was fed daily by oral gavage for 5 days. On day 5, after dosing, the mice were fasted for 24 h. On day 6, 30 min after the last dosing, the mice were fed BD 2000 solution.

As shown in Figure 1, thirty healthy male mice were randomly divided into 3 groups: control group (group I), domperidone group (group II), and ARE group (group III), with 10 mice in each group. Each group was fed distilled water (group I), the positive drug domperidone (5.0 mg/kg/d) (group II), and the AR water extract (2.0 g/kg/d) (group III) daily by oral gavage for 5 days. On the fifth day, after dosing, the mice were fasted without water restriction for 24 h. On the sixth day, the mice were fed BD 2000 solution (0.3 mL; 20 g/L) by oral gavage 30 min after the last dosing.

**Figure 2:**
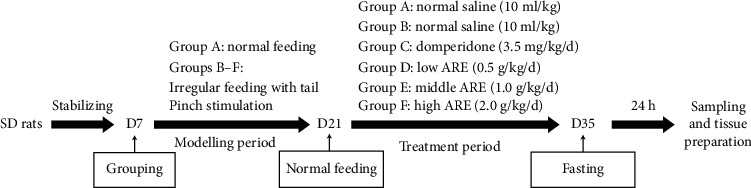
Except one, others were subjected to 14 days of tail-clamping stimulation to establish the FD rat model. Subsequently, 14 days of treatment was started and the corresponding treatment drugs were given to the different groups. The last administration of the drug was followed by a 24-hour fast and sampling was started.

As shown in Figure 2, after 7 days of stabilizing, sixty SD rats were randomly divided into 6 groups: blank group (group A), model group (group B), domperidone group (group C), ARE low-dose group (group D), ARE middle-dose group (group E), and ARE high-dose group (group F), 10 rats in each group. The groups were as follows: the rats in the control group were returned to normal feeding for 14 days. The other 5 groups were molded according to the “irregular feeding with tail pinch stimulation” method for 14 days.

Throughout the study, all rats were allowed free access to water. All rats had a normal water supply. Groups B–F: single-day feeding, double day fasting, and their food intake was recorded. On fasting days, the end 1/3 of the rat's tail with long sponge pliers was clamped. They were made to scream and struggle, but only to the extent that the skin is not broken; they were stimulated and made angry and allowed to fight with other rats in the cage (any rats scratched in fights were treated with iodine to prevent infection). Stimulation was performed for 10 min at 1 h intervals, stopped after 4 h, and finished after 14 days.

After modeling, the rats were allowed to return to normal feeding. In the five experimental groups, one model control group was left untreated, receiving menstruum only (normal saline 10 ml/kg); other treatment groups received either a low (0.5 g/kg/d), middle (1.0 g/kg/d), or high (2.0 g/kg/d) dose of the ARE and the positive drug domperidone (3.5 mg/kg/d) daily for 14 days. After this period, the general symptoms of rats, such as appearance, behavior, glandular secretion, and breathing, were observed. The bodyweight of each animal was recorded weekly, and the differences among groups were compared; the difference in daily food weight was used to calculate average food intake.

### 2.5. Gastrointestinal Motility as Indicated by BD 2000 [22]

After feeding BD 2000 for 20 min, the animals were sacrificed, and all the gastrointestinal tracts were excised. The gastric residue was fully dissolved with 2 mL distilled water and centrifuged at 3500 r·min^−1^ (15 min at 4°C), and the absorbance of the supernatant at a wavelength of 620 nm was measured. The mean absorbance of the blank control group was 100%. The ratio of the absorbance means of the treatment group to that of the blank control group was used as the gastric residual rate. The ratio of the distance from the pyloric sphincter to the pigmentary front and the distance from pigments to cecum was used as the intestinal propulsion rate.

### 2.6. MTL, GAS, VIP, and SS as Indicated by Enzyme-Linked Immunosorbent Assay (ELISA)

After the last dose, rats were fasted for 24 h. Rats were anesthetized with ether. Abdominal aortic blood was collected by laparotomy with 2 methods of blood collection, one with an empty pipe and another with the addition of 230 *μ*L 10% EDTA-Na and 30 *μ*L aprotinin enzyme. About 2 mL of blood was taken from the abdominal aorta. It was then centrifuged at a low temperature for 5 min at 2000 rev/min. The serum was separated and placed in a refrigerator at −20°C. Serum GAS was tested. Separate the plasma in a centrifuge tube with 10% EDTANa2 and aprotinin, store it in a refrigerator at −20°C, and measure the plasma MTL, VIP, and SS according to the ELISA kit procedure.

### 2.7. GAS and SS Expression of Antrum and Hypothalamus as Indicated by Immunohistochemistry

After the rats were killed, their brains were fixed for 48 h in a 10% formalin solution, and the hypothalamus was extracted. The stomach was removed at the same time, cut open, and flushed with 0.9% saline to remove any residue. Then, a portion of the stomach was removed and fixed in 10% formalin solution. Then, the antral tissue and hypothalamus were fixed with routine paraffin using routine procedures, and 4 *μ*m sections were stained with hematoxylin and eosin (HE).

The sections were stained for cellular localization and then processed using SP immunohistochemical staining under a microscope. Brown and dark yellow-brown particles in the membrane or cytoplasm were considered indicative of positive results. Using IPP image analysis software mining maps and image analysis, an area density (AD) map was constructed to indicate expression levels. The expression of GAS and SS in the hypothalamus and antral expression was determined.

### 2.8. VIP Receptor mRNA Levels as Indicated by Semiquantitative Reverse Transcription Polymerase Chain Reaction (RT-PCR)

The RNA sample was consistent with the amount of content transferred, and the analysis was performed in accordance with the instructions included in the one-step RT-PCR kit. The reaction reagent system had a total volume of 50 *μ*L, with the following reaction conditions: 30 cycles of reverse transcription at 50°C for 30 min, initial PCR denaturation for 2 min, denaturation at 94°C for 45 s, annealing at 59°C for 30 s, and 65°C for 1 min; this was followed by a 65°C final extension for 10 min. Five samples per experimental group were randomly selected. The *β*-actin RT-PCR method was performed as described above using the same method excepting that the annealing temperature was changed to 57°C. Then, the results of all reactions were evaluated under gel electrophoresis at a voltage of 100 V for 40 min. The results of gel imaging were photographed, and system software was used for optical density analysis of the PCR products. These were compared to the values for *β*-actin, and relative expression was calculated.

### 2.9. Statistical Analysis

Results are here expressed as the mean ± SD of the number (*N*) of mice in each experimental group. The overall significance of the results was determined by the one-way analysis of variance using the SPSS version 16.0 statistical software package. Probability values (P) below 0.05 were considered statistically significant.

## 3. Results

### 3.1. Effect on Gastric Emptying and Intestinal Propulsion

**Figure 3:**
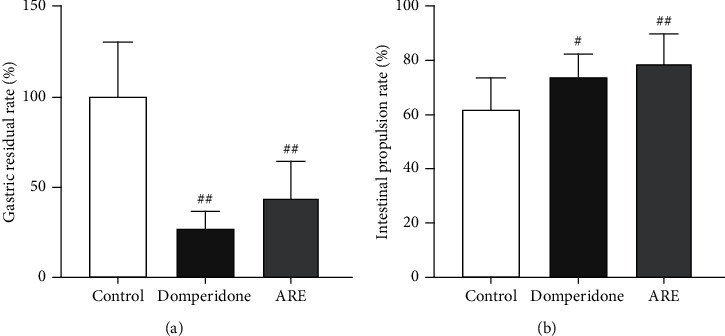
Effect of domperidone and ARE on gastric emptying and intestinal propulsion in mice. The data are expressed as the mean ± standard deviation (SD) (*n* = 10). (a) Calculated gastric residual rate. (b) Calculated intestinal propulsion rate. ^#^*P* < 0.05 versus the control; ^##^*P* < 0.01 versus the control.

As shown in Figure 3, compared with the blank group, after treated with AR, the gastric residual rate was significantly reduced, which meant that AR could improve the gastric emptying of mice (Figure 3(a)). Meanwhile, it also significantly improved the intestinal propulsion rate (Figure 3(b)). This was consistent with the results of the positive drug group and proved that AR could effectively improve the gastrointestinal motility of mice.

### 3.2. General Observations

Before modeling, all rats were normal in appearance, behavior, breathing, and so on. After modeling, the model group was observed for abnormal symptoms such as irritable temper, often tussling with each other, and scratching in limbs and head. Then, they looked for reduced activity, listlessness, hair without light, unresponsiveness, and thin feces. Finally, after intragastric administration for 14 days, drug-treated groups were observed for normal symptoms such as an increase in mean weight, normal drinking water, and normal daily behavior.

### 3.3. Changes in Weight and Food Intake in the Experiment

**Figure 4:**
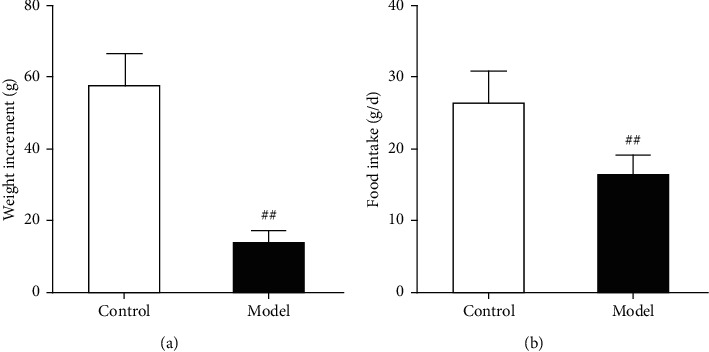
Changes in modeling on weight and food intake in rats. The data are expressed as the mean ± standard deviation (SD) (*n* = 10). (a) The amount of weight gain; (b) change in food intake. ^##^*P* < 0.01 versus the control.

**Figure 5:**
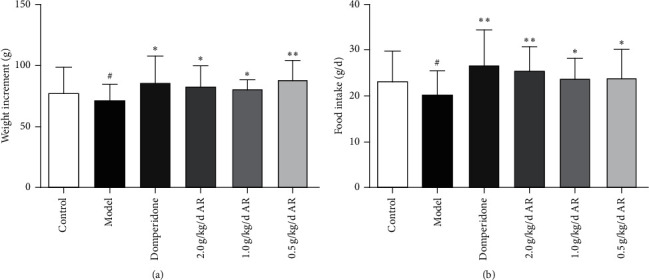
Effects of 14-day treatment on weight and food intake in rats. The data are expressed as the mean ± standard deviation (SD) (*n* = 10). (a) The amount of weight gain in each group. (b) Change in food intake in each group. ^#^*P* < 0.05 versus the control; ^*∗*^*P* < 0.05 versus the model group; ^*∗∗*^*P* < 0.01 versus the model group.

The changes in weight and food intake are shown in Figures 4 and 5. During the modeling period, the normal control rats gained weight significantly faster than those of the model group (Figure 4(a)). The part of rats in the model group showed weight loss. During the drug administration period, on average, compared with the normal group, the positive group and all the ARE group were significantly improved in weight (Figure 5(a)). During the modeling period, the rats in the model group ate significantly less than those in the normal control group (Figure 4(b)). During the drug administration period, the rats in the treated group ate significantly more than those in the model control group. This was true in both the positive drug group and all the ARE group; even the positive drug group and the high-dose ARE group showed the most pronounced difference (Figure 5(b)).

### 3.4. Changes in Levels of Gastrointestinal Hormone

**Figure 6:**
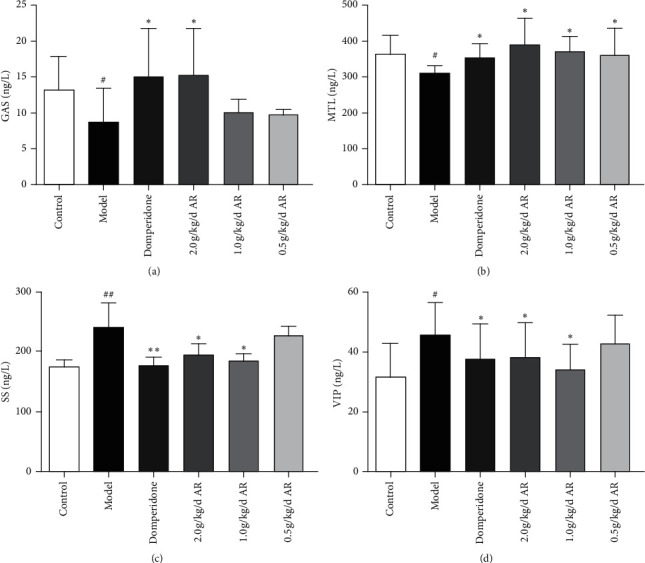
Effects of levels of gastrointestinal hormone. The data are expressed as the mean ± standard deviation (SD) (*n* = 10). (a) Serum levels of gastrin in each group. (b) Plasma levels of motilin in each group. (c) Plasma levels of SS in each group. (d) Plasma levels of VIP in each group. ^#^*P* < 0.05 versus the control; ^##^*P* < 0.01 versus the control; ^*∗*^*P* < 0.05 versus the model group; ^*∗∗*^*P* < 0.01 versus the model group.

The influence of this treatment on the gastrointestinal hormone is shown in Figure 6. Rats in the model group showed significantly lower serum levels of GAS than normal control rats, and the ARE dose group and positive drug group showed especially high levels (Figure 6(a)). Plasma MTL levels were significantly lower in the model group than in normal controls, but they were significantly higher in the ARE dose group and positive drug group (Figure 6(b)). The levels of plasma SS were significantly higher in model rats than in normal rats, but they were significantly lower in the ARE group and positive drug domperidone group after oral administration (Figure 6(c)). Plasma VIP levels were significantly higher in the model group than in normal controls but significantly lower in the ARE group and positive drug group after oral administration (Figure 6(d)).

### 3.5. Expression of GAS and SS in Gastric Antrum and Hypothalamus

**Figure 7:**
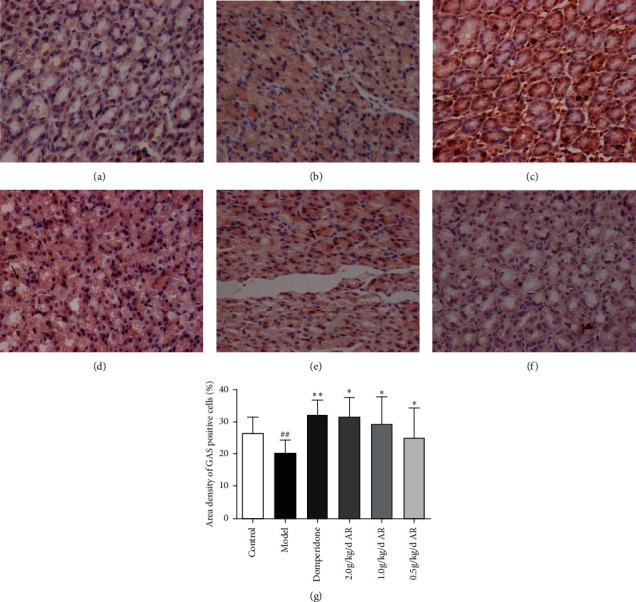
The expression of GAS in the gastric antrum tissue of rats detected by immunohistochemistry. Magnification, 400x; bars = 50 *μ*m. (a) Control group; (b) model group; (c) domperidone group (3.5 mg/kg); (d) AR extract (2 g/kg); (e) AR extract (1 g/kg); (f) AR extract (0.5 g/kg); (g) statistics of area density of positive cells. ^##^*P* < 0.01 versus the control; ^*∗*^*P* < 0.05 versus the model group; ^*∗∗*^*P* < 0.01 versus the model group.

**Figure 8:**
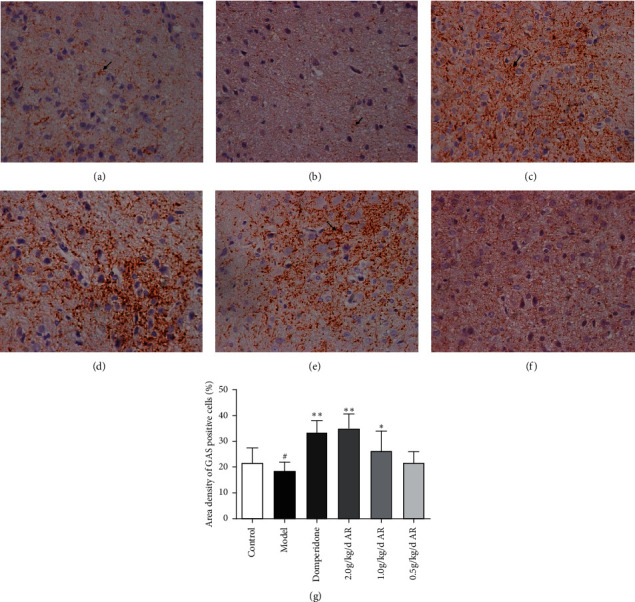
The expression of GAS in the hypothalamus tissue of rats detected by immunohistochemistry. Magnification, 400x; bars = 50 *μ*m. (a) Control group; (b) model group; (c) domperidone group (3.5 mg/kg); (d) AR extract (2 g/kg); (e) AR extract (1 g/kg); (f) AR extract (0.5 g/kg); (g) statistics of area density of positive cells. ^#^*P* < 0.05 versus the control; ^*∗*^*P* < 0.05 versus the model group; ^*∗∗*^*P* < 0.01 versus the model group.

**Figure 9:**
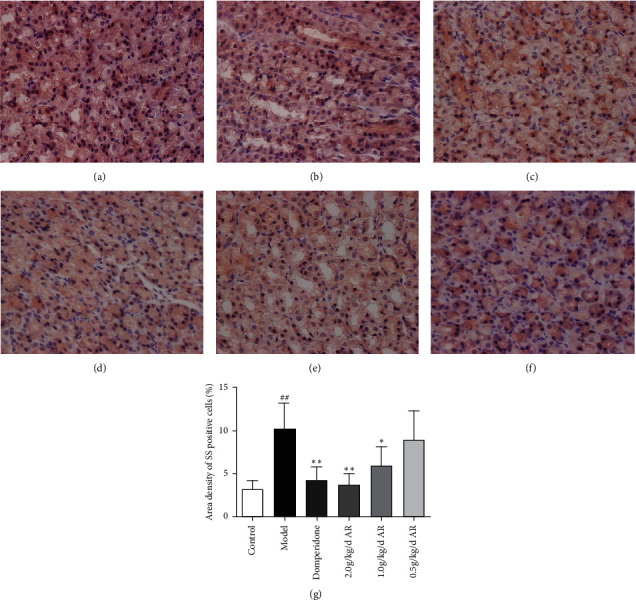
The expression of SS in the gastric antrum tissue of rats detected by immunohistochemistry. Magnification, 400x; bars = 50 *μ*m. (a) Control group; (b) model group; (c) domperidone group (3.5 mg/kg); (d) AR extract (2 g/kg); (e) AR extract (1 g/kg); (f) AR extract (0.5 g/kg); (g) statistics of area density of positive cells. ^#^*P* < 0.05 versus the control; ^##^*P* < 0.01 versus the control; ^*∗*^*P* < 0.05 versus the model group; ^*∗∗*^*P* < 0.01 versus the model group.

**Figure 10:**
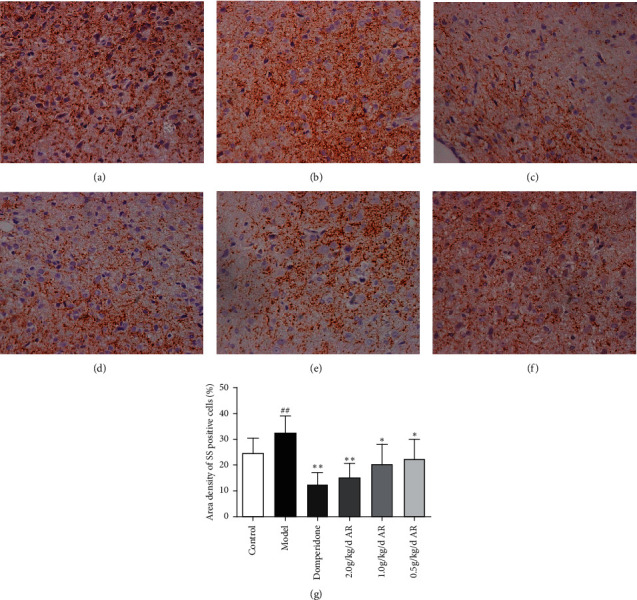
The expression of SS in the hypothalamus tissue of rats detected by immunohistochemistry. Magnification, 400x, bars = 50 *μ*m. (a) Control group; (b) model group; (c) domperidone group (3.5 mg/kg); (d) AR extract (2 g/kg); (e) AR extract (1 g/kg); (f) AR extract (0.5 g/kg); (g) statistics of area density of positive cells. ^#^*P* < 0.05 versus the control; ^##^*P* < 0.01 versus the control; ^*∗*^*P* < 0.05 versus the model group; ^*∗∗*^*P* < 0.01 versus the model group.

In the gastric antrum, GAS and SS are mainly expressed in the epithelial cells of the mucus layer; in the hypothalamus, GAS and SS are mainly expressed in the cytoplasm of neurons of the arcuate nucleus on both sides. As indicated by immunohistochemistry (Figures 7–10), ARE can enhance the expression of GAS in the antrum (Figure 7) and hypothalamus (Figure 8). This also reduces SS expression in the gastric antrum (Figure 9) and hypothalamus (Figure 10).

### 3.6. mRNA Expression of VIP in Antrum

**Figure 11:**
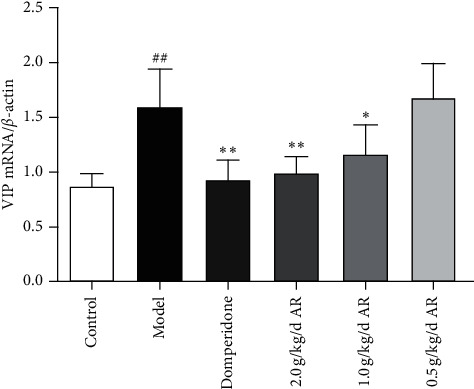
The relative expression of VIP mRNA/*β*-actin in the gastric antrum tissue of rats was measured by RT-PCR. ^#^*P* < 0.05 versus the control; ^##^*P* < 0.01 versus the control; ^*∗*^*P* < 0.05 versus the model group; ^*∗∗*^*P* < 0.01 versus the model group.

The results shown in Figure 11 indicate the effects of VIP on the relative mRNA expression of *β*-actin relative expression. ARE can inhibit VIP gene expression (*P* < 0.05) in model rats. The positive drug group and ARE group showed significantly less expression of VIP mRNA than the model group (*P* < 0.05 and *P* < 0.01).

## 4. Discussion

Commonly used drugs for the treatment of functional dyspepsia (FD) include acid suppressants, prokinetic agents, and anti-Helicobacter pylori drugs [23]. Domperidone is a peripheral dopamine receptor blocker that enhances gastric motility, promotes gastric emptying, and coordinates gastric and duodenal movements [24]. *Artemisia rupestris* L. is the perennial herb of rupestris belonging to Artemisia (Compositae), which is wildly distributed in Xinjiang (China), middle Asia, and Europe [25]. As a commonly used medicinal material for Xinjiang Uygurs and other ethnic minorities, AR has been shown to improve the symptoms of FD in clinical application. However, the key site of action and underlying mechanisms of AR are unclear. In the current study, we aimed to investigate the effects of AR on FD.

At present, studies have reported that nearly 50% of FD patients have gastrointestinal motility disorders such as gastroparesis, delayed gastric emptying, reduced exercise capacity, abnormal duodenal reflux, gastric movement dysfunction, and decreased gastric compliance [26]. Moreover, some gut hormones were abnormal in FD, which means brain-gut peptide may play an important role in the pathogenesis of FD [27]. For these reasons, we selected gastric emptying, intestinal propulsion, and four gastrointestinal hormones (GAS, MTL, VIP, and SS) to quantitatively assess the effects of AR.

In this study, the weight and food intake of the rats was measured and recorded before and after the FD rat model was established, as loss of food intake and weight is a clinical feature of FD [28]. The animal experiments showed that the weight and food intake of the rats in the model group was significantly reduced, which indicated that the rat model of FD was successfully developed. Following treatment, the weight and food intake of the rats in the rat model of FD increased in all treatment groups, and the mental state and physiological behavior also returned to normal. This is a preliminary proof that AR can improve FD symptoms.

Gastric emptying is the process of delivery of gastric contents into the duodenum. Through the propulsive effects of the stomach and duodenum [29], most FD patients have gastrointestinal motility disorders, which are related to delayed gastric emptying [12]. This study used dextran blue BD 2000 as a pigment marker to prove that ARE can promote the gastrointestinal motility function of mice, and there is no significant difference compared with domperidone.

GAS was secreted by the gastrin-releasing enteroendocrine cells (G-cells), which were found in the antrum and duodenum and regulated by central and peripheral nerves. The primary physiological function of gastrin is the stimulation of gastric acid secretion [30]; in addition, it can accelerate the gastric electric rhythm and promote gastric emptying [31]. Some experiments have indicated that rats with gastrointestinal disorders cause decrease in GAS in the plasma, gastrointestinal fluids, duodenum, gastric antrum, and tissues of the hypothalamus [32]. MTL is a polypeptide consisting of 22 amino acids, and its main role is in the stomach organ, where it aids the emptying of the stomach's contents. Apart from gastric emptying, motilin also influences the gallbladder and the lower esophageal sphincter. These contractile actions identify MTL as a gut-derived orexigenic peptide [33]. Experimental studies have shown that the fasting and postprandial plasma MTL values in the FD group were lower than those in the control group [34]. In our study, the GAS and MTL levels in the blood of FD rats were all reduced, and the area density of GAS positive cells in the hypothalamus and gastric antrum decreased significantly. After 14 days of treatment, the GAS and MTL blood levels of the treatment group were all increased, and the high-dose AR group was more significant. The expression of GAS also dramatically enhances in the hypothalamus and gastric antrum. This indicates that AR may relieve FD symptoms by enhancing the expression of GAS in the gastric antrum, hypothalamus, and the levels of MTL in plasma.

The peptide hormone SS can be found in gastric, pancreatic, and gastrointestinal nerves, posterior pituitary, and central nervous system, and it can inhibit the release of the part of gastric acid. SS can inhibit pepsin and gastrin-releasing hormone, and this strength of the symptoms and plasma levels of SS have been reported in patients with FD [35]. A clear correlation was reported between FD symptoms and plasma SS symptoms, such as this study has shown that fasting patients have significantly higher SS levels than normal controls, and the increase in SS is positively correlated with delayed gastric emptying [36]. Experimental studies have shown that food can not only stimulate the release of SS from the gastric antrum but also regulate the secretion of SS by stimulating CCK and the release of GAP and glucagon [32]. During the phase of digestion, the inhibitory effect of somatostatin on gastrin maintains gastric acid secretion at a relatively low level. During food intake, the inhibition of somatostatin by activating cholinergic neurons can maximize the secretion of gastric acid, while also directly stimulating G-cells [30]. VIP is an important gastrointestinal hormone and is widely distributed in the gastrointestinal tract and central nervous system, regulated to ion secretion, nutrient absorption, gut motility, glycemic control, carcinogenesis, immune responses, and circadian rhythms [37]. It has an inhibitory effect on the movement of the gastrointestinal tract, and it reduces the levels of GAS, MTL, and other gastrointestinal hormones [38]. The findings from the present study showed that plasma levels of SS and VIP in the rat model group were upregulated and downregulation of SS and VIP in the treated groups. The most significant reduction is in the middle-dose AR group. Semiquantitative RT-PCR analysis shows that AR extract can inhibit the VIP reverse transcription level, and this may be an important reason for the AR extract to treat gastrointestinal dysfunction.

Our experimental results show that AR can change the expression of BGP in the gastric antrum and hypothalamus to restore it to normal levels. This adjustment not only improves the gastrointestinal motility but also restores the mental condition to the state before FD symptoms appeared. We speculate that, under normal circumstances, the brain-gut peptide maintains a delicate balance in the regulatory pathway between the brain and the gastrointestinal tract, and any dysfunction of the brain-gut axis may disrupt this balance, resulting in physical and psychological abnormalities. Thus, AR may be used as a dose-related brain-gut axis modifier to improve FD symptoms through adjusting this balance. The pathogenesis of FD and the underlying pharmacological mechanism of AR will need further study, but it is certain that AR may render the treatment of FD more effectively. In the follow-up study, the pharmacodynamic study of compound preparations containing multiple herbal medicines will have a greater potential, and AR will be the most important component.

## 5. Conclusions

Our results demonstrated that ARE can effectively improve the gastrointestinal motility of FD model rats. Regulation of bidirectional expression of brain-gut peptides (GAS, MTL, SS, and VIP) may be a key mechanism.

## Figures and Tables

**Table 1:** Primer sequences of the genes selected for RT-PCR.

Gene name		Primers (5′–3′)	Product size	Tm
VIP	FW	5′-AAGGAAAGACCCAAGGAGGCA -3′	210 bp	61.33
	RV	5′-CATTCTCCGCTAAGGCATTCTG -3′		59.45
*β*-Actin	FW	5′-TGTGATGGTGGGAATGGGTCAG-3′	514 bp	62.28
	RV	5′-TTTGATGTCACGCACGATTTCC-3′		60.10

## Data Availability

The data used to support the findings of this study are available from the corresponding author upon request.
